# What Sexual and Gender Minority People Want Researchers to Know About Sexual Orientation and Gender Identity Questions: A Qualitative Study

**DOI:** 10.1007/s10508-020-01810-y

**Published:** 2020-09-01

**Authors:** Leslie W. Suen, Mitchell R. Lunn, Katie Katuzny, Sacha Finn, Laura Duncan, Jae Sevelius, Annesa Flentje, Matthew R. Capriotti, Micah E. Lubensky, Carolyn Hunt, Shannon Weber, Kirsten Bibbins-Domingo, Juno Obedin-Maliver

**Affiliations:** 1 The PRIDE Study/PRIDEnet, Stanford University School of Medicine, 1701 Page Mill Road, Palo Alto, CA 94304 USA; 2 Division of General Internal Medicine, Department of Medicine, University of California, San Francisco, CA USA; 3 Division of Nephrology, Department of Medicine, Stanford University School of Medicine, Stanford, CA USA; 4 Alliance Health Project, Department of Psychiatry, University of California, San Francisco, San Francisco, CA USA; 5 University of California, San Francisco School of Medicine, San Francisco, CA USA; 6 Division of Prevention Science, Department of Medicine, Center of Excellence for Transgender Health, University of California, San Francisco, San Francisco, CA USA; 7 School of Nursing-Community Health Systems, University of California, San Francisco School of Nursing, San Francisco, CA USA; 8 Department of Psychology, San José State University, San José, CA USA; 9 Department of Family and Community Medicine, University of California San Francisco, San Francisco, CA USA; 10 Department of Epidemiology and Biostatistics, University of California, San Francisco, San Francisco, CA USA; 11 Department of Obstetrics and Gynecology, Stanford University School of Medicine, Stanford, CA USA

**Keywords:** Sexual and gender minorities, Sexual orientation, Gender identity, Qualitative research, Health surveys

## Abstract

Sexual and gender minority (SGM) people—including members of the lesbian, gay, bisexual, transgender, and queer communities—are understudied and underrepresented in research. Current sexual orientation and gender identity (SOGI) questions do not sufficiently engage SGM people, and there is a critical gap in understanding how SOGI questions reduce inclusion and accurate empirical representation. We conducted a qualitative study to answer the question, “For SGM people, what are the major limitations with current SOGI questions?” Focus groups probed reactions to SOGI questions adapted from prior national surveys and clinical best practice guidelines. Questions were refined and presented in semi-structured cognitive interviews. Template analysis using a priori themes guided analysis. There were 74 participants: 55 in nine focus groups and 19 in cognitive interviews. Participants were diverse: 51.3% identified as gender minorities, 87.8% as sexual minorities, 8.1% as Hispanic/Latinx, 13.5% as Black or African-American, and 43.2% as Non-white. Two major themes emerged: (1) SOGI questions did not allow for identity fluidity and complexity, reducing inclusion and representation, and (2) SOGI question stems and answer choices were often not clear as to which SOGI dimension was being assessed. To our knowledge, this represents the largest body of qualitative data studying SGM perspectives when responding to SOGI questions. We present recommendations for future development and use of SOGI measures. Attention to these topics may improve meaningful participation of SGM people in research and implementation of such research within and for SGM communities.

**Electronic supplementary material:**

The online version of this article (10.1007/s10508-020-01810-y) contains supplementary material, which is available to authorized users.

## Introduction

Sexual and gender minority (SGM) people—including members of lesbian, gay, bisexual, transgender, and queer (LGBTQ) communities—make up approximately 4.5% of the United States population and face discrimination with worse health outcomes when compared to non-SGM populations (Ard & Makadon, 2012; Newport, 2018; The GenIUSS Group, 2014) (see Table 1 for definitions). SGM individuals are understudied and underrepresented in research, and accurate identification and inclusive data collection are critical to improving outcomes and reducing disparities (Cahill et al., 2014; Daniel, Butkus, & Health and Public Policy Committee of American College of Physicians, 2015; Institute of Medicine, 2011; Pérez-Stable, 2016). Recently, increasing national attention has been paid toward improving sexual orientation and gender identity (SOGI) questions in research and clinical settings to better identify SGM individuals (Badgett, 2009; Institute of Medicine, 2011, 2013; The GenIUSS Group, 2014). Improved SOGI questions not only have significant implications in data collection but also in bringing visibility to under-recognized communities (Spade, 2015). Accurately capturing SGM communities by using inclusive SOGI questions serves to validate and demarginalize their existences in society.

**Table 1:** Glossary of definitions

Term	Definition
Asexual	A sexual identity describing people who do not experience sexual attraction to people of any gender but may still have romantic attractions to other people
Aromantic	A romantic identity describing people who do not experience romantic attraction to people of any gender but may still have sexual attraction to other people
Bisexual	A sexual identity where sexual attractions and/or behaviors are focused on members of both sexes (usually female and male) or gender identities (women and men). Increasingly this is used to describe people whose sexual attractions and/or behaviors are with people of the same and/or another gender
Cisgender	A person with a gender identity the same as that commonly associated with their sex assigned at birth
Gay	A sexual identity where sexual attractions and/or behaviors are focused mainly on members of the same gender identity
Gender expression	Characteristics in a person’s appearance, personality, and behavior that are culturally and temporally defined as masculine, feminine, or outside of the masculine or feminine binary
Gender identity	A person’s deeply-felt, self-conceptualization of being a boy, a man, or male; a girl, a woman, or female; or another gender (e.g., genderqueer, gender nonconforming, gender neutral) that may or may not correspond to that commonly associated with a person’s sex assigned at birth or to a person’s primary or secondary sex characteristics
Gender minority	A person with a gender identity that differs from that commonly associated with their sex assigned at birth
Gender non-binary	A person whose gender identity does not fully fall along the gender binary of being a girl/woman or boy/man
Gender non-conforming	A person whose gender identity or expression does not fully conform to sex-linked social expectations (e.g., masculine girls/women, feminine boys/men)
Genderqueer	A gender identity usually used in one of two ways: (1) as an umbrella term that includes all people whose gender identity varies from the traditional cultural notions of gender; or (2) to describe a person whose gender identity does not fully fall along the gender binary of being girl/woman or boy/man, similar to gender non-binary
Graysexual/demisexual	A sexual identity where sexual attractions occur only occasionally and under specific circumstances, usually after developing a very strong bond
Heterosexual/straight	A sexual identity where attractions and/or behaviors are focused mainly on members of another gender identity
Intersex	A person who is born with any of a range of sex characteristics that may not fit typical notions of binary “male” or “female” bodies. Sometimes used to describe people who have differences of sex development
Lesbian	A sexual identity where attractions and/or behaviors are focused mainly on members of the same gender identity, usually referencing those who identify as women
Pansexual	A sexual identity where sexual attractions can occur toward individuals of all gender identities or expressions
Sex assigned at birth	The sex assigned to each person at time of birth or shortly thereafter usually based on external genitalia, also referred to as natal sex or biologic sex. This describes anatomic and/or physiologic characteristics
Sexual minority	A person with a sexual identity that is not strictly straight or heterosexual
Sexual orientation	An enduring pattern of emotional, romantic, and/or sexual attractions; a person’s sense of identity based on those attractions, related behaviors, and membership in a community of others who share those attractions
Transgender/trans	A person with a gender identity that differs from that commonly associated with their sex assigned at birth
Transgender man	A person who identifies as a man and was assigned female sex at birth
Transgender woman	A person who identifies as a woman and was assigned male sex at birth

Current SOGI questions are not sufficiently engaging SGM people. Testing and validation of new SOGI questions on a national level has largely focused on the perspectives of non-SGM individuals (Dahlhamer, Galinsky, Joestl, & Ward, 2013; Division of Health Interview Statistics, 2014; Miller & Ryan, 2011; Stern, Michaels, Milsei, Heim Viox, & Morrison, 2016). Further, recent SOGI questions have primarily added new answer choices to recognize underrepresented SGM identities (e.g., adding “transgender male” and “transgender female” as answer choices). However, this is done without critically examining how existing SOGI questions normalize cisgender and heterosexual identities while marginalizing non-normative SGM identities (Galupo, Davis, Grynkiewicz, & Mitchell, 2014; Westbrook & Saperstein, 2015). Specifically, national measurements fail to recognize key concepts for SGM communities: (1) sexual orientation (SO) and gender identity (GI) are independent aspects of identity and both can be dynamic, (2) self-conceptualization of one’s GI is not dependent on biologic sex assigned at birth and both GI and sex assigned at birth should be assessed, and (3) those who have a GI different from their sex assigned at birth are not a homogenous group that can be captured under the umbrella term of “transgender” (Hart, Saperstein, Magliozzi, & Westbrook, 2019; Lombardi & Banik, 2016; Magliozzi, Saperstein, & Westbrook, 2016; Westbrook & Saperstein, 2015). Suggested improvements to SOGI questions included using gradational questions of femininity and masculinity to reflect gender diversity among all individuals, or using new answer choices to recognize different communities previously categorized under the umbrella term of “transgender” (i.e., those who identify along the gender binary as either men or women versus those who identify outside of the binary such as genderqueer, genderfluid, or non-binary) (Cruz, 2014; Hart et al., 2019; Magliozzi et al., 2016). However, these proposed methods have not yet been rigorously tested or validated on a larger scale.

Few studies have examined SGM individuals’ perspectives on SOGI questions, and there is a critical gap in understanding the extent to which current SOGI questions lead to inaccurate or incomplete empirical reflections of this group. Using qualitative research to value input from these populations and to explore these complexities could provide opportunities to improve existing questions (Wallerstein & Duran, 2006; Willging, Salvador, & Kano, 2006). Focus groups are an effective first-step for improving research questions: (1) group interactions support creativity and idea generation, (2) participants can modify question terminology to enhance understanding, and (3) groups can create positive experiences between researchers and community members, rebuilding a sense of trust through collaborative design aligning research processes with community values (Krueger, 1994; Morgan, 1988; Nassar-McMillan & Borders, 2002; O’Brien, 1993). Individual cognitive interviews enhance this process by: (1) adding depth to concepts observed by focus groups, (2) supporting exploration of complex perspectives in individuals’ question understanding and interpretation, (3) allowing discussion between participant and researcher in a safe, confidential environment, and (4) strengthening construct validity of questions developed (Gill, Stewart, Treasure, & Chadwick, 2008; Willis & Artino, 2013).

We thus conducted a two-phase qualitative study of SGM individuals to answer the question, “For SGM people, what are the major limitations with current SOGI questions?” In Phase 1, we conducted focus groups among SGM identity clusters to explore perspectives when answering previously used and/or expert best practice recommendations for SOGI questions. In Phase 2, we used focus group discussion and themes to propose new SOGI questions and conducted cognitive testing interviews to delve deeper into how SGM participants engaged with these questions. By better understanding these processes, we hope SOGI questions can be improved to enhance comfort of SGM people during research participation, increase research engagement, and ensure scientific and conceptual rigor in the data collection process.

## Method

### Participants

We recruited self-identified SGM people aged 18 years or older who were able to speak, read, and write in English and resided in the United States. Participants were recruited from The Population Research in Identity and Disparities for Equality (PRIDE) Study (pridestudy.org), an existing online cohort of sexual and gender minority people, as well as from social media, community fliers, and LGBTQ community centers, reaching over 10,000 people (Lunn et al., 2019b). Potential participants completed a 22-item eligibility screening survey assessing current gender identity, sex assigned at birth, current sexual orientation, and other demographic questions (Supplemental Material A). Nearly 1,400 people filled out the screening survey online or during in-person recruiting, of which approximately 200 were eligible for in-person focus groups conducted in the San Francisco Bay Area, and approximately 1000 were eligible for remotely conducted cognitive interviews. We invited a set of approximately 200 eligible participants to focus groups or interviews to obtain a sample diverse in SOGI identities, sex assigned at birth, ethnic/racial backgrounds, ages, and (for cognitive interviews only) geographies. Recruitment for focus groups occurred from January to September 2016, and recruitment for cognitive interviews occurred from September 2017 to March 2018.

#### Focus Groups

We conducted nine in-person focus groups (approximately 2 h each) clustered around SGM identity. We conducted seven groups each clustered around a different SGM identity to maintain a level of homogeneity within groups and facilitate interactions between participants, including two groups of sexual minority women (including cisgender and transgender women), two groups of sexual minority men (including cisgender and transgender men), and three groups of gender minority people (gender minority men, gender minority women, and those who identified as gender fluid, gender non-conforming, or other gender non-binary identities) of various sexual orientations. We also conducted two groups with mixed SGM identities to ensure a diversity of perspectives (Acocella, 2012; Krueger, 1994; Morgan, 1988). Groups were mixed in terms of race/ethnicity, and five of the nine groups were majority participants of color. Focus groups had an average of six participants per group (range of three to nine), and the number of participants per group was largely determined by participant availability.

Focus group SOGI questions were chosen from prior national surveys and best practice recommendations and displayed at each focus group (Table 2). Questions from best practice recommendations were created and endorsed by groups of expert clinicians and researchers in SGM health research and policy (Badgett, 2009; Cahill et al., 2014; Reisner et al., 2014b; Sausa, Sevelius, Keatley, Rouse Iñiguez, & Reyes, 2009; The GenIUSS Group, The 2014), or came from national surveys that had completed extensive cognitive testing on specific SOGI questions among SGM and non-SGM participants (Dahlhamer et al., 2013; Division of Health Interview Statistics, 2014; Miller & Ryan, 2011; Ridolfo, Perez, & Miller, 2011; Ridolfo & Schoua-Glusberg, 2009). Using a semi-structured focus group guide, participants were introduced to the study’s aim of “helping researchers understand how to ask people about their sexual orientation and gender identity” and prompted to share their insights including limitations with question stems, answer choices, and things they would change (Supplemental Material B). Participants received a $30 gift card as compensation. SW facilitated all focus groups. As a researcher with experience in focus group facilitation especially among SGM populations, SW bolstered groups by intentionally gathering diverse perspectives and prevented monopolization of the discussion by individual participants. Eight focus groups were conducted at a LGBTQ community health center in San Francisco and one at the University of California, San Francisco from January 2016 to September 2016.

**Table 2:** Sexual orientation and gender identity (SOGI) questions used in focus groups

Gender identity question stems
How do you describe yourself? (The GenIUSS Group, 2014)
What is your current gender identity? (Cahill et al., 2014; Tate et al., 2013; The GenIUSS Group, 2014)
What is your sex or gender? (Lombardi & Banik, 2016; The GenIUSS Group, 2014)
What is your gender identity? (Callahan et al., 2015)
What is your sex or current gender? (Check all that apply) (Lombardi & Banik, 2016; Sausa et al., 2009; The GenIUSS Group, 2014)
Gender identity answer choices
Female
Male
Transgender, female to male
Other examples
Transgender male
Female-to-male (FTM)
Trans man
Transgender, male to female
Other examples
Transgender female
Male-to-female (MTF)
Trans woman
Genderqueer
Other examples
Genderqueer, neither exclusively male nor female
Gender non-conforming
Gender variant
Gender non-binary
Neutrois
Questioning/unsure
Other
Other examples
Other, please specify: [free-text field]
Additional category (please specify): [free-text field]
Additional gender category/(or Other), please specify: [free-text field]
Sex assigned at birth question stems
What sex were you assigned at birth, on your original birth certificate? (Bauer et al., 2017; Cahill et al., 2014; Institute of Medicine, 2013; Michaels et al., 2017; The GenIUSS Group, 2014)
What gender were you assigned at birth? (Tate et al., 2013)
What sex were you assigned at birth? (Cahill et al., 2014; Lombardi & Banik, 2016)
Sex assigned at birth answer choices
Male
Female
Sexual orientation question stems
Do you think of yourself as… (Cahill et al., 2014; The GenIUSS Group, 2014)
Do you consider yourself to be…(Badgett, 2009; Conron, Mimiaga, & Landers, 2010; Miller & Ryan, 2011; Ridolfo et al., 2012; VanKim, Padilla, Lee, & Goldstein, 2010)
Whether or not you are currently sexually active, what is your sexual identity or orientation? Please choose one answer. (Diamant, Wold, Spritzer, & Gelberg, 2000)
Which of the following best represents how you think of yourself? (Sell, 2007)
What is your sexual orientation? (Callahan et al., 2015; Ridolfo et al., 2012)
How do you currently identify your sexual orientation? (Katz-Wise, Reisner, Hughto, & Keo-Meier, 2016)
Sexual orientation answer choices
Straight or heterosexual
Gay, lesbian, or homosexual
Bisexual
Queer
Pansexual
Asexual
Question/unsure
Other: [free-text field]

#### Cognitive Interviews

Using focus group analysis and feedback, we created a list of cognitive interview SOGI questions, which included three question sets—each assessing SO, GI, or sex assigned at birth—with two to four questions per set. During ~ 45-min cognitive interviews, participants received an online survey displaying the cognitive interview SOGI questions (Table 3). Using a semi-structured cognitive interview guide, participants were told the goal of the interview was to “get your perspectives on the experience of interacting with research questions” and probes queried SOGI question and answer choice clarity, ease of understanding, comfort with answering, and emotional response (Supplemental Material C). Because interviews were conducted online, participants were required to have telephone and Internet access. Interviews were conducted by three trained research assistants with 17 interviews using BlueJeans online videoconferencing software (BlueJeans Network; San Jose, CA) and two interviews by telephone while the participants accessed the online surveys. Participants received a $20 gift card as compensation. Cognitive interviews took place from October 2017 to May 2018.

**Table 3:** Sexual orientation and gender identity (SOGI) questions used in cognitive interviews

Gender identity question stems
What is your gender?
What is your gender identity?
What is your current gender?
What is your current gender identity?
Gender identity answer choices
Female
Male
Woman
Man
Transgender female (MTF)
Transgender male (FTM)
Transgender woman (MTF)
Transgender man (FTM)
Gender non-conforming
Questioning/unsure
Other: [free-text field]
Decline to state
Sex assigned at birth question stems
What sex were you assigned at birth?
What sex were you assigned at birth, meaning on your original birth certificate?
Sex assigned at birth answer choices
Female
Male
Intersex
Decline to state
Sexual orientation question stems
What is your sexual orientation?
Which of the following best represents how you identify?
Regardless of your sexual experience, what is your sexual identity or orientation?
How do you currently identify your sexual orientation, regardless of if you are sexually active?
Sexual orientation answer choices
Straight, heterosexual
Straight
Heterosexual
Lesbian, gay, same-gender attraction
Lesbian
Gay
Same-gender attraction
Bisexual
Queer
Pansexual
Asexual, demisexual, graysexual
Questioning/unsure
Other: [free-text field]
Decline to state

### Qualitative Analysis

All focus groups and interviews were audio- and/or video-recorded, professionally transcribed, and coded using Dedoose (Version 7.0.23; SocioCultural Research Consultants, LLC; Manhattan Beach, CA; [Dedoose, 2016]). Attribution of participant characteristics collected from the initial screening survey with each quote followed the order of age, race(s)/ethnicity(ies), gender identity(ies), sex assigned at birth, sexual orientation(s), and respective focus group or cognitive interview. Quotes also included self-reported answers for race/ethnicity, gender identity, and sexual orientation. We utilized template analysis, a method of using a priori themes around sexual orientation, gender identity, and assigned sex at birth to guide coding and textual data analysis (Brooks, McCluskey, Turley, & King, 2015). Two researchers coded the focus groups, and three researchers coded the cognitive interviews. Focus group coding was completed using an a priori established codebook and modified iteratively after focus groups to encompass emergent domains from June to August 2017. The cognitive interview codebook was adapted from the focus group codebook and modified iteratively to account for variability between interviews. Coding for cognitive interviews took place from February to June 2018. For focus groups and interviews, coding was compared and reconciled until qualitative inter-coder consistency was established. For focus groups, thematic saturation was reached after nine focus groups and after 19 interviews for cognitive interviews. Application of codes from codebooks was clearly defined to ensure accuracy and consistency.

The diverse perspectives of the study investigators allowed for robust analysis of results. The majority of study investigators, including the lead authors, have various SGM identities. Although the SGM status of investigators was unknown to participants during data collection, the investigators’ familiarity with the history and experiences of SGM people likely encouraged an open and honest discussion. All investigators are university-affiliated researchers focused on SGM health outcomes, and lead authors of the study are also physicians. As such, our perspectives within health care and research settings as SGM clinicians, researchers, and patients bolstered the research by seeing our own life experiences reflected in the results. A smaller group of investigators, including the lead author, identify as people of color, and all authors actively engaged in discussions around the impact of SGM identities and race/ethnicity in the context of this work. This study received approval from the University of California, San Francisco Institutional Review Board.

## Results

There were 74 participants in the study: 55 in focus groups and 19 in cognitive interviews. The median age was 34 years (range of 20–72 years) (Table 4). For GI, 51.3% of participants were gender minority people, 21.6% identified as genderfluid, genderqueer, non-binary, or gender non-conforming, 71.6% as either man or woman, 29.7% as either transgender man or transgender woman, and 43.2% had more than one gender identity. For sex assigned at birth, 44.6% were assigned female at birth, and 54.1% were assigned male. For SO, 87.8% were sexual minority people, 16.2% identified as bisexual, 48.6% as lesbian or gay, 13.5% as straight or heterosexual, 35.1% as having more than one sexual orientation, and 47.3% as another sexual orientation. Participants were racially and ethnically diverse: 13.5% identified as African-American, 8.1% as Hispanic or Latinx, and in total, 43.2% identified as Non-White. During focus groups and cognitive interviews, participants often did not differentiate between medical and research settings, and for many of them, these settings were often interchangeable. Two major themes emerged: (1) SOGI questions did not allow for identity fluidity and complexity, reducing inclusion and representation, and (2) SOGI question stems and answer choices were often not clear as to which SOGI dimension was being assessed (Table 5).

**Table 4:** Participant demographics of focus group and cognitive interview participants assessing the responses of SGM people to questions on sexual orientation and gender identity (SOGI)^a^

	Focus groups (9 groups, *n* = 55 people)	Cognitive interviews (*n* = 19)	Total (*n* = 74)
Age (Median, IQR)	34 years (30–50)	33 years (27–52)	34 years (28–50)
18–29	13 (23.6%)	6 (31.6%)	19 (25.7%)
30–39	18 (32.7%)	5 (26.3%)	23 (31.1%)
40–49	9 (16.4%)	3 (15.8%)	12 (16.2%)
50–59	10 (18.2%)	2 (10.5%)	12 (16.2%)
≥ 60	3 (5.5%)	3 (15.8%)	6 (8.1%)
Gender identity (GI)			
Genderfluid, genderqueer, non-binary, or gender non-conforming	9 (16.4%)	7 (36.8%)	16 (21.6%)
Man	17 (30.9%)	7 (36.8%)	24 (32.4%)
Transgender man	4 (7.2%)	2 (10.5%)	6 (8.1%)
Transgender woman	14 (25.5%)	2 (10.5%)	16 (21.6%)
Woman	22 (40.0%)	7 (36.8%)	29 (39.2%)
Multiple gender identities	25 (45.5%)	7 (36.8%)	32 (43.2%)
Another gender identity	4 (7.3%)	1 (5.3%)	5 (6.8%)
Gender minority^b^	28 (50.9%)	10 (52.6%)	38 (51.3%)
Sex assigned at birth			
Female	22 (40.0%)	11 (57.9%)	33 (44.6%)
Male	32 (58.2%)	8 (42.1%)	40 (54.1%)
Sexual orientation (SO)			
Bisexual	7 (12.7%)	5 (26.3%)	12 (16.2%)
Lesbian or gay	29 (52.7%)	7 (36.8%)	36 (48.6%)
Straight or heterosexual	8 (14.5%)	2 (10.5%)	10 (13.5%)
Multiple sexual orientations	17 (30.9%)	9 (47.4%)	26 (35.1%)
Another sexual orientation	23 (41.8%)	12 (63.2%)	35 (47.3%)
Sexual minority^c^	47 (85.5%)	18 (94.7%)	65 (87.8%)
Race/ethnicity			
Asian or Pacific Islander	16 (29.1%)	2 (10.5%)	18 (24.3%)
Black or African-American	6 (10.9%)	4 (21.1%)	10 (13.5%)
Hispanic or Latinx	6 (10.9%)	0 (0%)	6 (8.1%)
White	29 (52.7%)	13 (68.4%)	42 (56.8%)
Multiple races/ethnicities	7 (12.7%)	3 (15.8%)	10 (13.5%)
Another race/ethnicity	4 (7.2%)	3 (15.8%)	7 (9.5%)
Educational attainment			
High school degree or less	5 (9.1%)	0 (0%)	5 (6.8%)
Some college	8 (14.5%)	6 (31.6%)	14 (18.9%)
Trade, technical, or vocational training	3 (5.5%)	1 (5.3%)	4 (5.4%)
4-year college degree	20 (36.4%)	7 (36.8%)	27 (36.5%)
Master’s degree or higher	16 (29.1%)	5 (26.3%)	31 (41.9%)
Declined to state	3 (5.5%)	0 (0%)	3 (4.1%)
Estimated annual income			
< $20,000	25 (45.5%)	6 (31.6%)	31 (41.9%)
$20,000–60,000	6 (10.9%)	6 (31.6%)	12 (16.2%)
$60,001–100,000	11 (20.0%)	4 (21.1%)	15 (20.3%)
> $100,000	9 (16.4%)	1 (5.3%)	10 (13.5%)
Declined to state	4 (7.3%)	1 (5.3%)	5 (6.8%)
Geography			
Midwest	–	5 (26.3%)	5 (6.8%)
Northeast	–	1 (5.3%)	1 (1.4%)
South	–	3 (15.8%)	3 (4.1%)
West	55 (100%)	10 (52.6%)	65 (87.8%)

^a^Summations of demographic categories may total more or less than 100% because participants were allowed to select more than one answer choice for each demographic question or could decline to answer

^b^Determined by investigators as any gender identity differing from that generally associated with sex assigned at birth

^c^Determined by investigators as any sexual identity that is not strictly heterosexual or straight

**Table 5:** Emergent themes from focus group and cognitive interview participants assessing the responses of SGM people to questions on sexual orientation and gender identity (SOGI), along with exemplar quotes

Emergent theme	Exemplar quote
Fluidity and complexity matter: SOGI questions did not allow for fluidity and complexity, reducing inclusion and representation	“When you ask a person their identity, I think instead of giving them boxes and labels to choose, I think the nicest thing would be is to put a line and let you put what you want your damn self. That would be the greatest thing in the world.” (51 years old, African American, woman/trans woman, assigned male at birth, straight, mixed focus group) “My own orientation has changed over time, you know? […] [Using the term ‘current’] is an acknowledgement that that’s a piece of my life. Like the question is validating. That, and saying ‘That’s okay. Like, you don’t have to be in a static box and we get that. Where are you at right now?’” (32 years old, White, woman/genderqueer or gender non-conforming, assigned female at birth, lesbian, cognitive interview)
SOGI dimension matters: SOGI question stems and answer choices were often not clear as to which SOGI dimension was being assessed	“I guess it depends on what the writers of the study are looking for. Wouldn’t it make more sense to at least break it down into, ‘What does your behavior include, past and present?’ or ‘What kinds of activities have you engaged in?’ And then if they want to know about your sexual orientation or your romantic orientation, that’s a different thing. So I would hope that they ask for what they actually want to know.” (24 years old, White, woman, assigned female at birth, lesbian/queer, sexual minority women focus group) “We kept mentioning how important it is to understand what they’re getting at. And I just think there is this critical difference between how you identify and what you do. And to hold those two things as both okay is a hard thing to accomplish without two questions that are really explicit.” (30, White, woman, assigned female at birth, lesbian, sexual minority women focus group)

### Sexual Orientation and Gender Identity Question Limitations: Reduced Inclusion and Representation

Participants emphasized that boundaries between identities for SO and GI are not clear cut. Because SO and GI exist on a spectrum, SOGI questions often failed to adequately capture the fluidity and complexity of SOGI identities. Participants overwhelmingly requested SOGI questions to capture and normalize SO and GI fluidity. Fluidity could be understood in two ways: (1) individuals’ identities may have multiple dimensions and vary between multiple identities at any one point in time (e.g., individuals who identify as genderfluid and do not view their GI as a static state), and (2) GI and SO can change over one’s lifetime (e.g., a person who identifies as one SO or GI earlier in life but identifies as another later in life). Participants suggested by adequately capturing the complexity and fluidity of SOGI identities, SOGI questions could lend themselves to being more inclusive and representative.

I think we definitely need room in health care for people to be other, more complicated things—that they were born one way and have undergone hormone treatments and are different, or their body has just developed in a way that doesn’t fit cleanly with sort of what you expect of the classic XX or XY body layouts. (38, White, genderqueer or gender non-conforming/indifferent to gender, assigned male at birth, bisexual/pansexual, genderqueer/gender non-binary focus group)

To acknowledge GI fluidity, many respondents wanted more non-binary and gender expansive terms (e.g., “gender non-conforming,” “non-binary,” “genderqueer,” “genderfluid,” “agender,” “bigender,” and “two-spirit”). When cognitive interview participants were probed if “gender non-conforming” could be replaced with “gender non-binary,” they preferred to include both answer choices as they represented identities from separate communities.

The answers are very binary. Like I didn’t feel at all included as a non-binary person. The only option that like allowed not being either a binary woman or male, or man or female, was “gender non-conforming” and that’s absolutely not the same thing as “genderqueer” or “non-binary.” (25, African American, non-binary, assigned female at birth, bisexual/queer, cognitive interview)

Gender non-conforming can be anybody. Because gender non-conforming just talks about how we present in our culture. And how we can be perceived by the way we present in culture. Gender non-binary may be pointing to something that people perceive as physiological within themselves…So, they’re different concepts. I would put both [“gender non-conforming” and “gender non-binary” as options]. (56, White, transgender woman, assigned male at birth, pansexual, cognitive interview)

Many participants also disagreed with using the terms “female-to-male,” “FTM,” “male-to-female,” “MTF,” etc. These terms read as if transition only occurred in one direction along the gender binary, oversimplified GI, and did not adequately acknowledge how identities could be fluid.

I mean, it feels like “F” or “M” seems like endpoints, but rather, it’s not endpoints for folks. Sometimes, it may be helpful to just ask what hormones you’re taking, or I guess “M to non-binary” or something else. (22, Asian or Pacific Islander, genderqueer/gender non-binary/cozy femme, assigned female at birth, queer/aromantic/asexual, genderqueer/gender non-binary focus group)

So, I think the “MTF,” “FTM” thing also reads as a little bit older. It’s terms that I think younger people especially don’t seem to use and even older people who are more recently transitioning don’t really use as much. It was really popular in the 90s or the 2000s, but nowadays I’d say it’s fallen out of use. (31, White, woman/transgender woman, assigned male at birth, lesbian, cognitive interview)

To capture SOGI complexity, participants wanted the option to choose more than one answer for both SO and GI questions. Cognitive interviews probed about preference for “select all that apply” versus “choose one best answer,” and participants overwhelmingly preferred the former. This simple step acknowledged a “one best answer” may not capture the complexity of individuals’ SOGI and could misrepresent their identities and/or experiences.

I feel like [“select all that apply”] would be more inclusive, and also, in a way, more representative for a lot of people. Otherwise, if it’s [“choose one best answer”], it feels like it’s so much more limiting, and it wouldn’t give a complete picture. (42, Asian or Pacific Islander, transgender man/gender non-conforming, assigned female at birth, straight/queer, cognitive interview)

If I were specifically asked at the end of that list to choose just one best answer, that I would have trouble choosing—because I absolutely identify as queer, and I’m attracted to all sorts of people, but I do also absolutely identify as asexual. So, it’s hard for me to separate those two, because I can’t imagine being only one or the other. (33, American Indian or Alaskan Native/White, gender non-conforming, assigned female at birth, bisexual/queer/asexual cognitive interview)

Most participants strongly desired a write-in answer choice for both SO and GI questions to give space for those who did not find themselves in listed answer choices and to provide representation and inclusion for under-recognized SGM communities. This sentiment resonated especially with participants of color in cognitive interviews who historically did not see their communities represented on surveys.

Having an option to write in your own response is empowering, creates opportunity to give voice to a community that has not been listened to and is really, really diverse community, with, you know, folks coming up with their own words for who they are. (34, Asian or Pacific Islander, man/transgender man, assigned female at birth, straight/queer, sexual minority men focus group)

There was agreement against including terms like “homosexual” and “heterosexual” in SO questions, as they were viewed as “too clinical” and never would be terms participants would use to describe themselves. These labels led to interpretations of discrimination and stigma, reminiscent of when homosexuality was considered a mental health disorder.

No one uses homosexual. And it has a very negative connotation to us…Because we’re in a very religious, conservative area and it gets used to hurt us all the time. (56, White, transgender woman, assigned male at birth, pansexual, cognitive interview)

I just really dislike the term “homosexual,” ‘cause it’s again, same thing with heterosexual, it feels so clinical and other-y. (20, American Indian or Alaskan Native/White, woman, assigned female at birth, bisexual/queer, cognitive interview)

In summary, SGM participants expressed a desire for both SOGI questions stems and answer choices to adequately capture both the complexity and fluidity of their SOGI identities and doing so could significantly increase inclusion and representation of SGM communities in research studies.

### Sexual Orientation and Gender Identity Questions: Dimensions Matter

In both focus groups and cognitive interviews, participants stressed the importance of understanding which SO or GI dimension was being assessed when answering SOGI questions. Namely, SO questions could be asking about any one of the SO dimensions including sexual behavior, sexual and romantic attraction, or internal sense of sexual identity. GI questions could be asking about GI dimensions including gender self-identification, sex assigned at birth, current and/or past anatomy, gender presentation/expression, and gender perceived by others. Questions that were not clear in what dimension they were assessing (e.g., “How do you describe yourself?” for SO) could lead to different interpretations, even when answer choices were presented to guide participants.

Realizing, you know, it depends on the setting, but is this about who you’re sleeping with or is this about how you identify? And is this about how you’re going to relate to the person who is speaking to you and how they speak to you about your life? Or is this, like, they just need to know who you’re fucking. (49, Asian or Pacific Islander, man, assigned male at birth, gay, sexual minority men focus group)

It is potentially a little vague if that question just comes out of nowhere. Like, there would need to be some sort of lead-in, because if someone just randomly was like, “How would you describe yourself?” I might be tempted to say, “I’m kind.” (30, White, woman, assigned female at birth, lesbian, mixed focus group)

Many SO question stems from best practice recommendations did not use the term “sexual orientation” but instead used non-specific wording (e.g., “Do you think of yourself as…”) (Table 2). In response, many participants emphasized the importance of asking multiple questions, each covering a single SO dimension. This included questioning the use and meaning of the term “sexual orientation,” which was differently understood by different participants, and emphasizing how sexual behaviors could not be assumed or known based on only knowing a person’s sexual orientation.

Separate out the questions that you’re actually asking. If you’re asking about behavior, ask about behavior. And if you’re asking about orientation, ask about orientation. […] So, you can break it down a number of different ways, but you do need to break it down, especially depending on what you’re getting at. (24, White, woman, assigned female at birth, lesbian/queer, sexual minority women focus group)

It’s confusing, because behavior doesn’t really associate with your identity […] So, then it’s like okay if you’re asexual and you do have sex with someone of whatever gender, then like, [the identity] doesn’t really say anything. (23, Asian or Pacific Islander, woman, assigned female at birth, queer/asexual/panromantic, mixed focus group)

Most understood the phrase “sexual orientation” as referring to one’s self-conceptualization of sexual identity. However, the phrase “sexual orientation” was preferred in question stems over “sexual identity” (e.g., “What is your sexual orientation?” instead of “What is your sexual identity?”) because the former was more familiar and direct.

I’m familiar with the question [“What is your sexual orientation?”]. No one on the street asks me, “What do you identify as?” They ask me, “What is your sexual orientation?” It’s kind of like, “What is the color of your skin?” That’s simple. But if you were to ask me, “What do you culturally identify as?” I’d be like, “Well, jeez, I don’t know.” Most people have these very clear-cut cultural identities. Okay, I’m white. And this is the norm. But then, I’m biracial, so I’d have to get into, you know, “Well, what really is considered biracial? What percent this, and what percent that?” Whereas, like, the question [“What is your sexual orientation?”] is just kind of like a baseline question. So, it’s comfortable. (27, biracial White/African American, woman, assigned female at birth, bisexual/queer, cognitive interview)

When the phrase “Regardless of your sexual experience…” was included in SO questions, participants interpreted this as a cue the question was asking about identity and not behavior. Many in focus groups and cognitive interviews preferred this wording because it emphasized SO was more than just sexual behavior. Further, many participants indicated that the “Regardless of your sexual experience….” phrasing could be helpful for those not sexually active, who identify as asexual, and who have behaviors or attractions not traditionally corresponding with their identified orientations (e.g., lesbians who have sex with men).

I like that it says the part about whether or not you’re currently sexually active or not. Both for people who are questioning or considering their own identity, and also it emphasizes that it’s about the person and not about the partners. (31, White, woman, assigned female at birth, queer, sexual minority women focus group)

When you talk to non-LGBTQ individuals and you talk about who you are, what you identify as, it’s almost immediately, like, about sex. And just for that question to say, right off the bat, “Regardless of sexual activity…” that makes you feel comfortable, because we’re taking away that phrase that you dread that you know that you’re going to get. (27, biracial White/African American, woman, assigned female at birth, bisexual/queer, cognitive interview)

Because some people may not have a partner or be abstinent—currently abstinent for whatever reason, you know, medical, or emotional, or just had a sexual assault or something, but they still have a sexual orientation. (42, Asian or Pacific Islander, gender non-conforming/woman, assigned female at birth, bisexual/queer, mixed focus group)

Questions assessing GI prompted similar concerns about question clarity. Most participants understood “gender” to suggest one’s self-conceptualization of identity while “sex” referred to physical anatomy or sex assignment at birth. However, many mentioned that “gender” and “sex” were often used interchangeably. This conflation required additional clarity of what GI domain was actually being assessed.

Like the question, ‘What is your gender?’ Well, then you get into, that depends on who you’re frickin’ talk to […] Do you wanna know what genitalia I have? What hormones are flowing through my body? What the heck? You know? It’s a much more complicated answer. [laughing] (54, White, man, assigned female at birth, straight, cognitive interview)

For me, like the question [“What is your sex or gender?”], it’s conflating sex and gender and it’s asking a single question about both. It’s like, okay, which of these do you actually want to know about? Which of them do you care about for the context we are working in? Like, because they are potentially different things. (38, White, genderqueer or gender non-conforming/indifferent to gender, assigned male at birth, bisexual/pansexual, genderqueer/gender non-binary focus group)

When the term “gender identity” was included in the question stem, for some, it suggested an interest in self-identification rather than external perceptions, physical anatomy, or other domains. This was clearer and more specific than saying “gender” alone.

It’s tricky. Jesus Louise. Because you asked me, “What is my gender?”, and my response is “male.” But then I see all the other choices [like “transgender male”], and I’m like, “Oh. Well, do they wanna know this other information for some reason.” Like, [laughs] do you know what I mean? “Cause I don’t identify as transgender. Like I mean I know a lot of people that do. And they do because they’re activists and stuff. And it’s important to them to be seen as trans. But I was always male. The fact that I didn’t appear that way to the outside world for a long time, a lot of that was the outside world’s fault. […] Like I’ve been in medical situations where I had to figure out ahead of time was it significant that I still have female plumbing. Like is that medically necessary when I’m getting a colonoscopy? […] I have to know a lot more about medicine than any normal human being should have to know. I feel like, [for the question, “What is your gender identity?”], that one I answered, “man” to. That was easier to answer. Because now I know what you’re asking. You’re asking me how do I identify. Okay. That I can tell you. (54, White, man, assigned female at birth, straight, cognitive interview)

Participants of transgender experience in both focus groups and cognitive interviews echoed uncertainty about how to answer—“What is your gender?”—especially if it was unknown whether sex assigned at birth would later be assessed. They felt conflicted about answering with their gender identity (e.g., man or woman) instead of transgender man or woman. Many of them did not identify as transgender, but they also wanted to communicate their transgender experience or history in case the information was relevant. This conflict was particularly echoed by several African-American women of transgender experience, especially in focus groups where participants of color were the majority. They reported being less likely to identify with a “transgender” label than their White counterparts, leading to consideration of the intersectionality of SGM identity and race/ethnicity.

I think having the options “male,” “female,” “trans male,” and “trans female” together is very confusing because a lot of trans people do not identify with the label “trans,” so then they would check “male” or “female.” But then they don’t know what your purpose is in asking. […] So, I don’t know if you can do this on a one-part question. I would ask two questions or three even. (30, Hispanic/Latinx and White, genderqueer or gender non-conforming, assigned female at birth, asexual, genderqueer/gender non-binary focus group)

I’m a woman. I want people to respect me as a woman. I’m not no transgender. Well, I am a transgender, but I don’t like to go by transgender. (33, African American/Asian Pacific Islander, woman, assigned male at birth, bisexual, mixed focus group)

In summary, participants preferred questions that were specific and clear in stating which SOGI dimension was being assessed.

Not to like, short circuit the whole thing, but I feel like the question that really ought to be asked is, “What do we as medical practitioners or researchers need to know about your sex and gender identity in order to provide appropriate care?” Like, that’s the question that all of these are trying to get at. (38, White, genderqueer or gender non-conforming/indifferent to gender, assigned male at birth, bisexual/pansexual, genderqueer/gender non-binary focus group)

## Discussion

To our knowledge, this study represents the largest body of qualitative data exploring diverse perspectives of SGM individuals and SOGI question limitations. Two major themes emerged: (1) SOGI questions did not allow for identity fluidity and complexity, reducing inclusion and representation, and (2) SOGI question stems and answer choices were often not clear as to which SOGI dimension was being assessed. With these findings in mind, we developed recommendations for future SOGI measure development and use in research (Table 6).

**Table 6:** Recommendations for sexual orientation and gender identity (SOGI) questions

For all SOGI measures
Questions should allow, acknowledge, and normalize fluidity and complexity of people’s identities
SOGI questions should attend to cultural, regional, and linguistic variations between racial and ethnic groups
SOGI answer choices should be conceptualized as nominal categories, similar to race/ethnicity to better attend to diversity and complexity of identities
Include write-in answer choices in whatever domain of SO or GI is being assessed
Participants should have the option to select more than one answer choice and be prompted to do so with “select all that apply”
For sexual orientation (SO) measures
When feasible, assess all three dimensions of sexuality including attractions, behaviors, and identity(ies)
Be specific about the dimension of SO being assessed, specifically as it refers to sexual attraction, romantic attraction, sexual behavior, or internal sexual self-identification
Provide a diverse range of responses for SO questions, including asexual, pansexual, queer, and fluid
For gender identity (GI) measures
Provide a diverse range of responses for GI questions, including gender non-conforming, non-binary, and genderfluid
Be specific about the dimension of GI being assessed, specifically as it refers to gender expression, current/prior anatomy, birth sex assignment, or internal GI
Use a two-step approach of including one question to assess current GI and a second question to assess sex assigned at birth
Display both GI and sex assigned at birth questions simultaneously and/or include introductory text that notes both GI and sex assigned at birth will be assessed

### Implications and Recommendations for Sexual Orientation and Gender Identity Questions

Having SOGI questions address the complexity and fluidity of SOGI identities was important to participants so that researchers and health professionals could increase inclusion and improve the accuracy of empirical representations of these groups. Historically, SOGI questions have been rooted in conceptualizations of SO and GI as linear continua, with SO categories existing as a point on the continuum between heterosexuality and homosexuality, and GI categories as on a continuum between strictly man and woman identities (Kinsey, Pomeroy, & Martin, 1948; Kinsey, Pomeroy, Martin, & Gebhard, 1953). The majority of our participants rejected the conceptualization of identity as existing along a linear continuum (Galupo et al., 2014). Participants noted that requiring participants to identify as a singular point on a continuum did not acknowledge those with fluid/multiple SGM identities or identities existing outside of the binary of heterosexual/homosexual and man/woman. Instead, we recommend conceptualizing SO and GI categorizations as nominal categories (similar to conceptualizations of race and ethnicity) with each separate group having a rich historical, cultural, and political context.

In our study, participants frequently discussed how their SGM identities intersected with their social, cultural, and political identities to influence responses to SOGI questions. This was especially true for participants of transgender experience. African-American women of transgender experience noted how their communities would be less likely to openly adopt the “transgender” label to describe themselves and would only do so if relevant to medical contexts. This may be due to safety concerns given the high rates of discrimination and violence experienced by transwomen of color, wanting to increase internal gender affirmation, and/or to avoid stigma associated with “not passing” (Sevelius, 2013). Future work should consider exploring the role of intersecting identities through the lens of intersectionality, a concept that underscores how relationships between a person’s many social identities—including race/ethnicity, gender identity, sexual identity, and disability status among others—need to be examined within the context of interacting systems of inequality and oppression (Collins, 1998; Crenshaw, 1991). Researchers can use this concept to elucidate how traditional SOGI questions operate under assumptions that normalize and privilege heterosexual, cisgender, White, and able-bodied identities, and thereby cast SGM, non-White, and disabled identities as non-normalized and thus more invisible (Galupo et al., 2014; Hines, 2010). Researchers can then re-imagine how SOGI questions can more comprehensively capture the full range of SGM identities and experiences, and work toward eradicating structures of oppression that have rendered SGM communities invisible for so long (Richman & Zucker, 2019; Turan et al., 2019).

Based on our findings, we make two further recommendations: 1) allowing multiple answers with the prompt “select all that apply” in question stems, and 2) including a write-in option to acknowledge SOGI fluidity and complexity (Sausa et al., 2009; The GenIUSS Group, 2014). Researchers historically have found implementing these methods difficult for SOGI questions, where the numbers of participants using a write-in or “other” option were usually small and heterogenous. The heterogeneity of these results made drawing statistically meaningful conclusions difficult, and researchers often discarded these data (Ridolfo, Miller, & Maitland, 2012). SOGI terms also have different meanings when translated from English to other languages. Depending on the translation and cultural variations, participants could be self-categorizing themselves differently or using the write-in option different from originally intended when SOGI questions are translated into different languages (Badgett, 2009; Bauer, Braimoh, Scheim, & Dharma, 2017; Reisner et al., 2014a). These challenges led to prior recommendations against use of a write-in option because of discarded results and the assertion that SGM people would choose one of the provided options if no “other” category exists (Badgett, 2009).

Our findings, however, suggest that lack of a write-in options and the opportunity to “select all that apply” could reduce engagement and lead to potential miscategorization. In The PRIDE Study (pridestudy.org) pilot of over 16,000 SGM people in the United States, 2.5% selected “another gender identity,” 5.9% selected more than one gender identity, 4.3% chose “another sexual orientation,” and 16.8% chose more than one sexual orientation (Lunn et al., 2019a). In a study of sexual minority people responding to SO questions from the National Health Interview Survey, 7% of sexual minority respondents surveyed answered as “something else” compared to less than 1% of the general population (Eliason, Radix, McElroy, Garbers, & Haynes, 2016). With such significant numbers of SGM individuals answering as “another gender identity” or “another sexual orientation,” omitting the write-in option may fail to identify a sizeable portion of SGM individuals from within general population samples, thereby mischaracterizing outcomes for specific SGM communities. Use of a write-in response option may also allow researchers to track how language used by SGM communities evolves over time and may identify frequently occurring but not yet standardized identities (Eliason et al., 2016; Harrison, Grant, & Herman, 2011; Mayer et al., 2008). In a similar vein, racial categories and communities of color were historically lumped together which normalized White identities. Only by bringing out the importance of these nominal categories and allowing space for people to be “something else” in SOGI can we start to see the difference in people’s experiences and identities. We strongly recommend the use of “select all that apply” (or similar language) along with the write-in option to enhance SGM engagement whenever within the goal and scope of the study.

### Implications and Recommendations for Sexual Orientation Questions

Researchers have long cited how SO is comprised of several dimensions: sexual behavior, sexual and romantic attraction, and sexual identity, all of which may be dynamic over an individual’s lifetime (e.g., identify as gay earlier in life and later identify as bisexual), and individuals may have fluid or multiple sexual orientations at any one point in time (Diamond, 2003; Laumann, Gagnon, Michael, & Michaels, 1994; Sell, 2007). Additionally, all three SO dimensions may not be congruent for an individual (Diamond, 2003; van Anders, 2015). For example, a cisgender man may identify as bisexual because of his sexual and/or romantic attraction to people of other gender identities though may have sex only with other men.

Current best practices recommend, when feasible, measuring all three SO dimensions to increase sensitivity in identifying sexual minority individuals and allow for more comprehensive data collection. However, most studies have focused on the single measure of sexual orientation identity (Badgett, 2009; Patterson, Jabson, & Bowen, 2017; Wolff, Wells, Ventura-DiPersia, Renson, & Grov, 2017). When probed about specific SO questions obtained from current best practice recommendations such as “How do you describe yourself?” (Badgett, 2009; Ridolfo et al., 2011; Ridolfo & Schoua-Glusberg, 2009), participants disliked ambiguity and did not know which SO dimension was assessed even if answer choices were presented. We recommend precisely stating which SO dimension is being assessed and to assess multiple dimensions of SO when feasible. If assessing sexual identity, we recommend the question assess for “sexual orientation.” Interestingly, the recommendation contradicts prior best practice recommendations to use non-specific SO questions (e.g., “Do you consider yourself to be:”) and to avoid the term “sexual orientation” in the question stem due to limited translations for “sexual orientation” when translated into other languages (Badgett, 2009). Because our study only included perspectives of English speakers, content validity of questions when translated into other languages must be further evaluated.

Prior studies noted how sexual minority participants interpret questions asking about “sexual orientation” and “sexual identity” differently, despite both terms traditionally being used to assess one’s sexual self-identification (Galupo et al., 2014; Worthington & Reynolds, 2009). “Sexual identity” may be more specific in referring to one’s self-conceptualization of their sexual orientation. However, our results found participants more often preferred questions asking about “sexual orientation” rather than “sexual identity” because the former was more direct, familiar, and thus easier to answer. Respondents stated adding a clause to explicitly distinguish identity from behaviors (e.g., “Regardless of your sexual experience…”) was desirable. We propose the SO question—“Regardless of your sexual experience, what is your sexual orientation?”—as a possible question stem addressing these concerns and grounded in our findings.

As previously discussed, prior SO questions and their responses have traditionally been rooted in conceptualizations of all sexual identities along a heterosexual/homosexual continuum and fail to address how identities can be fluid. Our findings support the growing body of literature indicating a continuum approach is inadequate as the experiences of sexual minority people are often lost in this description. To address this, we recommend the approach of using more expansive SO response choices to include additional answer choices for asexual, pansexual, fluid, and queer. We combine this approach with methods above to acknowledge identity fluidity to ensure sexual minority people can be more accurately described and served by research studies.

### Implications and Recommendations for Gender Identity Questions

Assessing gender in research poses several epidemiological challenges given the multiple dimensions defining gender including gender identity, gender expression, gender perceived by others, and sex assigned at birth (Tate, Ledbetter, & Youssef, 2013). Prior studies have experimented with deconstructing GI by using gradational measures to assess femininity/masculinity scales to allow for fluidity instead of discrete answer choices (Westbrook & Saperstein, 2015). However, using these scales may be too grounded in the gender binary (Galupo et al., 2014). Other studies have underscored the importance of recognizing communities who were historically considered under the umbrella term of “transgender” as actually at least two groups: 1) those who identify along the gender binary (e.g., man, woman) despite their sex assigned at birth and 2) those who exist outside of the gender binary (e.g., genderqueer, non-binary) (Cruz, 2014; Hart et al., 2019). We agree with this recommendation and recommend adding non-binary gender response options (e.g., gender non-conforming, non-binary, genderqueer) to elucidate the nuances and differences between these groups and to ensure conceptual rigor (Conron, Scout, & Austin, 2008; Deutsch et al., 2013; Harrison et al., 2011; Kuper, Nussbaum, & Mustanski, 2012; The GenIUSS Group, 2014). In population-level surveys, we recognize implementing an exhaustive list of choices poses practical challenges. Careful consideration of the study population and meaningful gender identity responses based on the research question should guide the measures used.

Participants overwhelmingly disapproved of GI question stems lacking clarity on which gender dimension was being assessed. Simply asking about “gender” or “sex” of an individual was unclear as these terms were often conflated and could refer to any of the GI dimensions. A clearer question stem (e.g., “What is your gender identity?”) allowed the participant to avoid misclassification or communication errors and conveyed GI was being assessed. We recommend applying this question to all SGM and non-SGM research participants to avoid this stigmatization, to ensure accurate characterization of participants, and to normalize its use in all surveys. This finding is consistent with several prior best practice recommendations (Michaels et al., 2017; Reisner et al., 2014a; Stern et al., 2016; The GenIUSS Group, 2014).

In addition, current best practices recommend the two-step gender identity method (i.e., one question for internal gender identity, one question for sex assigned at birth) to accurately identify gender minority people (Reisner et al., 2014a; Sausa et al., 2009; Tate et al., 2013; The GenIUSS Group, 2014). Using two questions differentiates cisgender individuals from individuals who may identify differently from their sex assigned at birth but do not adopt the “transgender” label. This method was more sensitive in identifying gender minorities than a single question (i.e., one-step) approach and had reliable comprehension and acceptability among non-SGM and SGM populations (Lombardi, Banik, Mitchell, & Zuber, 2013; Reisner et al., 2014b; Stern et al., 2016; Tate et al., 2013). Despite this, the two-step approach has not been largely adopted in research and health surveillance studies (Bauer et al., 2017; Patterson et al., 2017; Wolff et al., 2017). Our findings align with prior studies supporting the two-step approach, as many participants who did not identify as their sex assigned at birth discussed not using or variably using the “transgender” label. In addition, we recommend presenting both questions together to guide respondents toward accurate answer choices.

Similar to how participants favored adding the phrase “regardless of your sexual experience” to SO question stems, a similar clarification can be considered for GI question stems. One proposed example is: “Regardless of your sex assigned at birth, what is your gender identity?” This question should be paired with a second question—“What is your sex assigned at birth?”—to utilize the two-step approach, and the two questions should be displayed simultaneously if possible. This method acknowledges and normalizes possible differences between one’s sex assigned at birth and internal GI. It clarifies that internal GI is the dimension of interest allowing participants to differentiate their gender identity from their sex assigned at birth if necessary without requiring them to adopt the “transgender” label. As we did not explicitly test the question “Regardless of your sex assigned at birth, what is your gender identity?” with participants, further investigation of this question prior to adoption is warranted.

### Strengths, Limitations, and Next Steps

In our study, we sought to gather a wide range of perspectives from individuals with various SGM identities to inform the development and deployment of SOGI measures for research. Major strengths include being the largest qualitative study to our knowledge attempting to answer the question, “For SGM people, what are the limitations with current SOGI questions?”; using purposeful sampling to create a sample diverse in SGM identities, age, educational attainment, socioeconomic status, and race/ethnicity; and using Internet and telephone tools to reach a wider geographic area away from urban and SGM-enriched areas.

Study limitations included only having U.S.-residing, English-speaking participants. This is especially important as prior studies have documented challenges when translating SOGI questions into other languages (Michaels et al., 2017; Reisner et al., 2014a; Ridolfo et al., 2011). Further work is needed to assess whether these findings are applicable in other languages and socio-linguistic and cultural contexts. Cognitive interviews were conducted online, requiring Internet and telephone access, which may bias toward participants of a higher socioeconomic status. Although participants were probed on their perspectives of answering SOGI questions in research contexts, participants often did not distinguish between medical and research contexts but spoke generally to how SOGI questions could be posed in either setting. This may be due to the fact that for the majority of SGM people participating in research, the settings in which they receive medical care and participate in research are often one in the same, and that the majority of SGM health research occurs either within medical contexts and/or is related to their experiences receiving medical care. This speaks to how the clinical and research realms can and often do overlap, underlining the importance for both communities to ensure appropriate care.

We envision future work that includes testing and development of SOGI questions using recommendations provided here including using quantitative iterative testing among larger sample sizes of SGM and non-SGM people to measure response validity and to assess participant understanding and acceptability.

## Electronic supplementary material

Below is the link to the electronic supplementary material.

Supplementary material 1 (DOCX 45 kb)
