# Supplementary material for: What Sexual and Gender Minority People Want Researchers to Know About Sexual Orientation and Gender Identity Questions: A Qualitative Study
Source: Arch Sex Behav. 2020 Sep 1;49(7):2301–18. doi: 10.1007/s10508-020-01810-y (PMC7497435; doi:10.1007/s10508-020-01810-y)
Supplement: Supplementary file 1 — Supplementary material 1 (DOCX 45 kb) [file 10508_2020_1810_MOESM1_ESM.docx]

**Supplemental Material A: Recruitment Screening Survey**

The PRIDE Study is a national health study of lesbian, gay, bisexual, transgender, queer (LGBTQ), and other sexual or gender minority (SGM) adults.

The PRIDE Study is being conducted by a team of researchers at the University of California, San Francisco, many of whom are a part of the LGBTQ community. The goal of The PRIDE Study is to improve the health of LGBTQ people. To do this, we will explore how being LGBTQ is related to all aspects of health and life.

We will study the unique strengths, resiliencies, and challenges of our communities. We will share our findings the LGBTQ communities and health professionals.

As a first step, we need to gather information about LGBTQ identities and experiences. To do this, we are launching **The Questions on Sexual Orientation and Gender Identity Project (Q-SOGI).**

The Q-SOGI Project will allow us to partner with members of the community to understand how to best collect sexual orientation and gender identity information for research.

You can help The Q-SOGI Project by participating in focus groups, interviews, or online surveys. **If you are LGBTQ or SGM, are 18 years or older, live in the United States, and can read and speak fluently in English,** please consider participating in the study by filling out the following eligibility survey.

In the survey, you may be asked some sensitive questions. The focus of which is to ensure a diverse pool of respondents in the study. All of the information you provide will be protected and kept confidential. This research has been approved by the UCSF Committee on Human Research.

1. I consent to be screened for the purpose of participating in The PRIDE Study Q-SOGI Project and I consent to have the answers to the following questions be used by The Q-SOGI Project and UCSF for research purposes.
   1. Yes
   2. No
2. What is your current age? (You must be 18 years or older to participate)
3. Please enter your zip code:
4. Are you able to read, write, and have a conversation fluently in English?
5. Yes
6. No
7. What is your current gender identity (choose all that apply):
   1. Man
   2. Woman
   3. Female-to-Male (FTM), Transgender Male, or Trans Man
   4. Male-to-Female (MTF), Transgender Female, or Trans Woman
   5. Genderqueer or Gender Non-conforming
   6. I don’t know / Currently questioning
   7. Additional Gender Category or Other, please describe:
   8. Decline to answer
8. Do you think of yourself as (choose all that apply):
   1. Lesbian, gay, or homosexual
   2. Straight or heterosexual
   3. Bisexual
   4. Queer
   5. Asexual
   6. I don’t know / Currently questioning
   7. Another identity, please describe:
   8. Decline to answer
9. What sex were you assigned at birth on your original birth certificate (choose one):
   1. Male
   2. Female
   3. Decline to state
10. Are you of Hispanic, Latino, or Spanish Origin? (choose one)
    1. Yes
    2. No
    3. Decline to state
11. What is your race? (check all that apply)
    1. White
    2. Black, African American, or Negro
    3. American Indian or Alaskan Native
    4. Other, please describe:
    5. Decline to answer
12. Which of the categories best describes your current annual income before taxes?
    1. $0 - 5,000
    2. $5,001 – 10,000
    3. $10,001 – 15,000
    4. $15,001 – 20,000
    5. $20,001 – 30,000
    6. $30,001 – 40,000
    7. $40,001 – 50,000
    8. $50,001 – 60,000
    9. $60,001 – 70,000
    10. $70,001 – 80,000
    11. $80,001 – 90,000
    12. $90,001 – 100,000
    13. $100,000+
    14. Decline to answer
13. What is your highest level of education completed (choose one)
    1. No schooling
    2. Nursery school to high school, no diploma
    3. High school graduate or equivalent (e.g. GED)
    4. Trade, technical, or vocational training
    5. Some college
    6. 2-year college degree
    7. 4-year college degree
    8. Master’s degree
    9. Doctoral degree
    10. Professional degree (e.g., M.D., J.D., M.B.A.)
    11. Decline to answer
14. How do you hear about The Q-SOGI Project? (select all that apply)
    1. Social media (e.g., Facebook post, Twitter)
    2. Email from community organization
    3. Community organization website
    4. Flyer from community organization
    5. Friend or family member
    6. Health care provider
    7. The PRIDE Study website
    8. Other, please describe:
15. From which community organization(s) did you hear about The Q-SOGI Project?
16. First name:
17. Last name:
18. Your pronouns (e.g., she/her, he/him, they/them, etc.):
19. Which of the following ways would you like to be contacted regarding follow-up information about the study (choose all that apply):
    1. Email
    2. Phone
    3. Text message
20. What is your email address?
21. What is your phone number (e.g., 415-555-1212)?
22. Is it okay to leave a message at this number about your involvement in The Q-SOGI Project?
    1. Yes
    2. No
23. Are you willing to attend in-person events such as focus groups or interviews in the San Francisco or East Bay areas?
    1. Yes
    2. No
24. If you are assigned to attend a focus group or interview, do you have a preference to attend in San Francisco or in Concord?
    1. San Francisco
    2. Concord
    3. I have no preference and can attend events in either San Francisco or Concord

**Supplemental Material B: Focus Group Facilitator Guide**

*(NOTE TO FACILITATOR: Read only bolded text. Additional text is provided to prompt ideas for probes, so read only as needed to guide the conversation.)*

**Brief intro: Thank you so much for coming before we start the focus group we are going to give you some information about what the plan is for today and go over some ground rules. Everybody ready to get started?**

**Discussion Format:**

**Today we will be doing a focus group. This means we will be holding a space to hear YOUR opinions about a topic. The focus group today is organized by The PRIDE Study and the goal today is help figure out about helping researchers understand about how to ask people about their sexual orientation and gender identity. We will talk more about this in a few minutes. We would like the discussion to be informal, so there’s no need to wait for us to call on you to respond. In fact, we encourage you to respond directly to the comments other people make. However, please do speak one at a time so we can all hear what is being said. If you don’t understand a question, please let us know. We are here to ask questions, listen, and make sure everyone has a chance to share. There are no wrong or right answers. As a reminder, we will be audio recording the discussion because we don’t want to miss any of your comments. No one outside of this room will have access to these recordings, and they will be destroyed after our report is written.**

**Verbal Consent:**

*(Read Informed Consent document. Allow 3-4 minutes after reading for additional questions).*

**By offering verbal consent, you have agreed to participate in a focus group to discuss this topic. We thank you in advance for your participation. Your thoughts are important for us to understand how to respectfully include lesbian, gay, bisexual, transgender, and queer (LGBTQ) people in research.**

**Participant Introductions:**

(*Team introductions)*

**Throughout the day we will be referring to the names on people’s name-tags and using the pronouns that are visible there. These will be noted in the recording but the public will never know your names. If you would like to have a different name used or if you didn’t get a name-tag, this is the time to take care of that now.**

**I’m going to ask each of you to introduce yourself to the group. Please only give a first name. Also, the name you choose to introduce yourself does not need to be the name you usually use. You can introduce yourself using any first name or alias you choose. Then please also state what pronouns you use to describe yourself.** *(Have participants introduce themselves by their first name only.)*

**Ground Rules:**

**Now I want to go over a few guidelines for the group discussion.**

1. **What we say in the group should stay here – keep it confidential.**
2. **One person talks at a time. Because we would like to hear from everyone, I might call on you if you’re not saying much, or ask you to give others a chance if you have talked a lot.**
3. **Feel free to respond to another group member, not just to my questions. You can follow up on what someone has said, agree or disagree, or give an example. Please speak for yourself using “I” statements and avoid attacking or putting someone else’s ideas down.**
4. **We’ll be talking some about gender identities, which can be a sensitive topic. It’s OK to talk about anything at all here. We really want to hear from you. If you prefer, you may talk about SGM people in general instead of your own experiences.**
5. **We want to hear a lot of different perspectives, so as a group, we have to respect everyone’s point of view. Everyone won’t agree, but we want to hear from everybody. There are no right or wrong answers! We want to ensure a safe environment where individuals speak openly so remember that everyone’s opinion and experience is valuable.**
6. **To protect everyone’s privacy, please use only the first names that we used to introduce ourselves.**
7. **Feel free to get up from the table at any time. You may want to get more snacks, go to the restroom, or just take a break or a stretch.**
8. **Please take a moment now to turn off all cell phones or place them on silent.** (Give them a moment now to do this.)

**Are there any ground rules that we should add to these?** (Give them a moment to discuss.)

**Can everybody work with these guidelines?** (Allow them to confirm.)

**Introduction to the Study:**

**Slide 1: Welcome to The PRIDE Study QSOGI Focus Groups. QSOGI stands for Questions on Sexual Orientation and Gender Identity. We’ll spend the next few minutes introducing the study and then we’ll start our focus group discussion. Please feel free to stop and ask questions at any point.**

**Slide 2: First, some terms that we all may or may not be familiar with. Members of the LGBTQ community, all fall under the umbrella of Sexual and Gender Minorities, shortened to SGM, or people who do not identify as heterosexual and/or cisgender. While this group may share the identity of <<*IDENTITY GROUP>>*, everyone also comes from different backgrounds, cultures, beliefs, and experiences and therefore might use different words to identify.**

**Slide 3: Sometimes people may think sexual orientation and gender are the same thing, but they’re actually quite different. To illustrate the point, we have the Gender Unicorn. The Gender Unicorn goes through all of the different aspects that go into someone’s identity, including their Gender Identity, Sexual Orientation/Attraction, Gender Expression, etc. Tonight we’ll focus on 3 things--Gender Identity, Sex Assigned at Birth, and Sexual Orientation. As you can see, Gender Identity is what’s inside a person’s brain and is whatever gender or no gender they see themselves as. These can be female, male, agender, transgender, etc. We will focus most on gender identity tonight. Sexual orientation is a complex identity of who someone may be sexually or romantically attracted to, so people can use the terms gay, lesbian, asexual, pansexual, etc. Some people use the shortcut gender identity is who you go to bed as whereas sexual orientation is who you go to bed with. This is not perfect, but it helps delineate the two concepts.**

**Slide 3: The LGBTQ or SGM community is underserved, understudied, and vulnerable.**

**Slide 4: Studies have that lesbian, gay, bisexual, transgender, and queer (LGBTQ) people have different health and healthcare needs than their heterosexual and cisgender counterparts. But due to a history of discrimination and lack of understanding, health care has largely failed meeting the needs of SGM communities. Here we see that 90% of transgender folks and 50% of LGB people feel like their health professionals were not adequately trained to take care of them. Part of that is because LGBTQ people are not included in research.**

**Slide 5: LGBTQ people are not routinely studied by large health policy and health research organizations. Without having LGBTQ people involved in research, it is hard to know exactly what the health and healthcare needs are and how to address them. The PRIDE Study was created to meet this need. It is one of the first national research studies asking the question, How does being a sexual and/or gender minority influence health?**

**Slide 6: (Show video)**

**Slide 7: One of the challenges to including LGBTQ people in research is knowing how to ask about sexual orientation, attraction, behavior, and gender identity in sensitive and meaningful ways. The purpose of the QSOGI Project is to find out what sexual and gender minority, or SGM, people think about various ways of asking about sexual orientation and gender identity to help us ensure that the way we ask these questions in research feels right and real. In order for research to be meaningful, we need to also be able to define identities that most accurately capture the complexities of our community. So that’s what we’re here to discuss today, is how do we make questions and answers to help researchers best ask SGMs about their identities? The Questions on Sexual Orientation and Gender Identity, or QSOGI, Project, is here to do that. The first part, the Focus Groups, is to hold discussions about what they like or don’t like about questions on sexual orientation and gender identity, and why, in order to develop questions that are informed and sensitive to the experiences of SGMs. After that, we’ll do in person interviewing and online testing to further refine the questions we develop.**

**Slide 8: Our goal is to leave this room with improved questions about gender identity, specifically about *IDENTITY GROUP* identity, sex assigned at birth and sexual orientation, all created as a collaboration by this group. As participants of the focus groups, you’re helping to shape how health workers can promote the health of SGMs, so thank you for participating today. Does anyone have any questions?**

**Okay, let’s begin. I will turn on the digital recorders now.**

*(TURN ON DIGITAL RECORDERS.)*

**Gender Identity/Sex Assigned at Birth Question Review**

**One of the challenges we’ve found in research about and for LGBTQ people is the lack of detailed information about people’s gender identities. So, to begin the conversation, let’s look at some ways to ask about gender identity. In your packet, please flip to the part where it says, “Gender Identity Question Stems” at the top. (***Give some time for participants to flip through packet and locate the page.)*

**I’ll read the instructions out loud. (***Read instructions on Question Review Packet.)* **We’re going to go through each of these question stems individually, and share feedback for each one. You’ll see boxes for each question, and those are for your reference only to take notes and to share your thoughts with us. You definitely don’t have to fill out each box. Please also share how you would edit the questions to make them better. We’ll be following along and writing everyone’s comments on the screen. (***Give participants time to let information sink in.)* **Does anyone have any questions? If not, we can begin.**

*(For each question stem and answer choice combination, we’ll ask these questions):*

**Question Stems – Gender Identity:**

- Do you see yourself and your community represented in the question? Why or why not?
- Was there anything unclear about the question?
- How could the question be improved?
- Are there any aspects of the question that you like?
- If you were given this question, how would you feel about it?
- If we were to create one question that best captures what everyone’s feedback, what would it be?
- Can we do a vote of whether or not we like these questions? (Show hand signals for upvote, downvote, and standby)
- Are there any ways in which you feel the question is weird or awkward?
- Are there any ways in which you feel the question is offensive?
- If you were faced with this question, would you answer it?

**Now that we’ve shared feedback on how we feel about the question, we’re going to vote based on what everyone has said. We encourage everyone to put in a vote for the question. The voting options are UPVOTE (***raise fist in air to denote upvote),* **a STANDBY option (**raise fist horizontally to denote standby) **if you are unsure about the question or would vote upvote the question if you could incorporate edits, and** **DOWNVOTE** *(raise fist to point downward with elbow up).* **Does anyone have any questions about the voting?** *(Go through voting process for the question, making sure to ask people who are standby why they voted that way).*

*Repeat question review and voting process for each question, and then move on to Answer Choice review*.

**Now, let’s move on to the answer choices. We’ll focus specifically on ANSWERS that describe the Gender Identity of <<*IDENTITY GROUP>>. (*** *Read instructions for Gender Identity Answer Choices section*.) **Again, feel free to write down your notes in the boxes provided about what you do or don’t like, but don’t feel the need to fill them all out. We’ll follow along on the screen with what people share.**

- Do you see yourself/ your community represented in the answers?
- Was there anything unclear about the answer choices?
- Are there any terms / answer choices that should be added?
- Are there any terms you would add or delete based on our previous list?
- How could the answer choices be improved?
- If you were faced with these answer choices, would you answer it?
- If you were given these answer choices, how would you feel about it?
- Are there any ways in which you feel the answers are weird or awkward?
- Are there any ways in which you feel the answer choices are offensive?
- Are there any aspects of the answer choices that you like?

**Great, that was a lot of information. Now let’s take a five-minute break to use the bathroom or get some light refreshments, and when we come back, we’ll discuss questions around gender identity and sex assigned at birth.**

**5 MINUTE BREAK (*TIME, X* minutes into group)**

**Okay, now let’s move on to the next question that’s usually paired with gender identity. The next question is about sex assigned at birth. Let’s all flip to the packet where it says, “Sex Assigned at Birth Question Stems”.**

**Notice we use the word, ‘assigned’, because this is the sex given right when we’re born and may not necessarily be a reflection of who we truly are. We ask this question also to respect those who have gender identities that may be different from their birth certificates but don’t see themselves as trans or another label. For example, someone can be born and assigned female, transition, and identify as male. Asking this second question captures their SGM status, but still respects their gender identity.**

**Again, feel free to write down your notes in the boxes provided for each answer choice about what you do or don’t like with what you would change, and we’ll follow along on the screen with what people share.**

*(Ask same block of questions as above regarding question stems and answer choices now for Sex Assigned at Birth)*

**Question Stems – Sex Assigned at Birth:**

- Do you see yourself and your community represented in the question? Why or why not?
- Was there anything unclear about the question?
- How could the question be improved?
- Are there any aspects of the question that you like?
- If you were given this question, how would you feel about it?
- If we were to create one question that best captures what everyone’s feedback, what would it be?
- Can we do a vote of whether or not we like these questions? (Show hand signals for upvote, downvote, and standby)
- Are there any ways in which you feel the question is weird or awkward?
- Are there any ways in which you feel the question is offensive?
- If you were faced with this question, would you answer it?

**Now, let’s move on to the answer choices. We’ll focus specifically on ANSWERS that describe the Sex Assigned at Birth of <<*IDENTITY GROUP>>.* Again, feel free to write down your notes in the boxes provided for each answer choice about what you do or don’t like with what you would change, and we’ll follow along on the screen with what people share.**

- Do you see yourself/ your community represented in the answers?
- Was there anything unclear about the answer choices?
- Are there any terms / answer choices that should be added?
- Are there any terms you would add or delete based on our previous list?
- How could the answer choices be improved?
- If you were faced with these answer choices, would you answer it?
- If you were given these answer choices, how would you feel about it?
- Are there any ways in which you feel the answers are weird or awkward?
- Are there any ways in which you feel the answer choices are offensive?
- Are there any aspects of the answer choices that you like?

**Sexual Orientation Question Review**

**Now we’ll go through various ways that people are asked about their sexual orientation. In your packet, please flip to the part where it says “Sexual Orientation Question Stems” at the top. (***Give some time for participants to flip through packet and locate the page.)*

**Question Stems – Sexual Orientation:**

- Do you see yourself and your community represented in the question? Why or why not?
- Was there anything unclear about the question?
- How could the question be improved?
- Are there any aspects of the question that you like?
- If you were given this question, how would you feel about it?
- If we were to create one question that best captures what everyone’s feedback, what would it be?
- Can we do a vote of whether or not we like these questions? (Show hand signals for upvote, downvote, and standby)
- Are there any ways in which you feel the question is weird or awkward?
- Are there any ways in which you feel the question is offensive?
- If you were faced with this question, would you answer it?

**Now, let’s move on to the answer choices. We’ll focus specifically on ANSWERS that describe the sexual orientation of *IDENTITY GROUP.***

- Do you see yourself/ your community represented in the answers?
- Was there anything unclear about the answer choices?
- Are there any terms / answer choices that should be added?
- Are there any terms you would add or delete based on our previous list?
- How could the answer choices be improved?
- If you were faced with these answer choices, would you answer it?
- If you were given these answer choices, how would you feel about it?
- Are there any ways in which you feel the answers are weird or awkward?
- Are there any ways in which you feel the answer choices are offensive?
- Are there any aspects of the answer choices that you like?

**Questions on Question Ordering**

**So now that we see all three questions and how they’re asked, let’s talk about the order of the questions.**

- Do you think it matters if we ask about sexual orientation or gender identity first?
- Do you think it matters if we ask about sex assigned at birth before gender identity?
- Do you think it matters if we ask about attraction or behavior first?
- What would be the easiest questions for you to answer?
- What would be the hardest questions for you to answer?
- Can you describe what makes a question hard or easy to answer?

**Other Questions if More Time (Optional):**

PRIDE Study Environment

**Finally, let’s talk a little bit about the question environment. All of these questions will be administered to people electronically on a computer, laptop, or mobile device. For some people, this may be a personal device, or it may be a shared one (work or library).**

- What do you think about this strategy for asking these questions?

- Do you think it will work to get information about LGBTQ people’s lives?

- Would you be willing to stay connected for years with a research study that was an online survey of no longer than 30 minutes at least once a year but maybe more?

- What would make you more or less willing to stay connected with the research study?

Recruitment:

- Where do you think the ideal places to recruit people for an online study would be?

- What websites do you generally use?

- What apps do you frequently use? Are any of them LGBTQ-focused?

- What social media sites do you visit?

- Do you go to web for health information? If so, what sites?

- What type of advertisement or electronic presence would get you excited about being involved?

- Would you participate in a study like this if you were given information about it by your doctor?

- Would you participate in a study like this if you were given information about it by a friend or community member?

- Do you have any ideas about how The PRIDE Study could reach people who don’t normally engage with an online study?

**Concluding Thoughts**

**When you think about whether you would enroll in a study like the one we are describing, what are the biggest factors that influence your thoughts about this?**

- Overall, what is your opinion about collecting sexual orientation and gender identity data for research?
- Do you have any general feedback on how today’s group went and what you’d like to change in the future? You can also write any further comments on an anonymous survey after if that makes you more comfortable

**Thank you very much for your time today. I would like to remind everyone that we have asked for everything that has been said today to be kept confidential. The information you have provided will be very helpful in helping us to think about the right ways to ask people about sexual orientation and gender identity in research and we hope will help improve the health of LGBTQ people.**

**Supplemental Material C: Cognitive Interview Guide**

*Note: Bolded questions/statements should be said to / asked of all participants, probe questions (not bolded) do NOT need to be asked of all participants and should only be used if needed to elicit participants’ perspectives on the central question.*

*Before beginning the interview make sure to take the following steps:*

1. *Confirm who it is you are interviewing – reviewing name, pronouns, and email address*
2. *Ensure that the tracking spreadsheet is up to date for the participant that you are about to interview*
3. *Open BlueJeans application*
4. *Open and prepare email to participant with the Qualtrics Survey link*

*15 minutes prior to the interview send the participant an email through Blue Jeans asking them to join the meeting at the previously agreed upon time.*

**Interview Intro:**

**Thank you again [*participant name*] for taking the time to participate in this study.**

**As we discussed, the goal of the interview is to get your perspective on the experience of interacting with the research questions we’ve designed and in particular your experience around answering questions about sexual orientation and gender identity.**

**By sexual orientation I mean there will be questions about how you identify with respect to your sexual attraction, behavior, and or identity. Common sexual orientation terms lesbian, gay, bisexual, transgender, queer, heterosexual or straight, or another sexual orientation. By gender identity, I mean your internal sense of your gender. This may manifest as an internal sense of being a man, a woman, or another gender. For many people, their internal gender may be different than that generally associated with the sex they were assigned at birth. So, we will be asking about the sex you were assigned at birth as well as your current gender identity.**

*[Review Informed Consent with participant, pausing along the way to allow the participant to voice questions or concerns.]*

*[Pause for a moment here to let participant take this in.]*

**Do you have any questions before we begin the interview?**

*[Press record on the right side of the screen. Pause for BlueJeans to announce that recording has begun]*

**Ok, I’ve enabled the recording option, and we’ll begin the interview.**

*[Send participant email with the interview survey]*

**I’d like you to complete a survey asking about gender identity and sexual orientation. You will find the link to the survey in an email that I’ve just sent to you. Please confirm that you have received the email. I’d like you to complete the survey while paying attention to how filling out each question made you feel and whether or not these questions made sense to you. I will not have access to any of your answers. Please begin the survey now and let me know when you have completed all the questions.**

[*Wait while the participant takes the survey*.]

**SECTION 1: Ease of understanding sexual orientation questions and answer choices**

*Goals: To ascertain the participant’s understanding of question phrasing, question and answer choice vocabulary, and instructions.*

**Now that you have completed the survey, I’d like to discuss your reaction to the question stems and answer choices. As we go through each question, I would like to hear any thoughts, questions, reactions, or critiques you had while taking the survey. Do you have any questions before we begin this next step in the interview?**

**First, we’re going to review the sexual orientation question stems and then we’ll review the sexual orientation answer choices. For each question, please share your reactions when you read the question. What did this question stem call to mind? You can also tell me what you like, dislike, or any changes you would make. We’ll go through each question stem independently.**

Sexual Orientation Question Stems:

1. What is your sexual orientation?
2. Which of the following best represents how you identify?
3. Regardless of your sexual experience, what is your sexual identity or orientation?
4. How do you currently identify your sexual orientation, regardless of if you are sexually active?

**Was there any confusion when you were answering any of these questions?**

1. *If there was any confusion, ask what was the source of the confusion. Were there different interpretations of the question that would change their answer?*
2. Did all of the words make sense to you? Did you understand the meaning of all of these words?

*If no, determine which words are unclear. Ask participant what they think the word or phrase means*.

**Between the four questions, which did you prefer when asked about sexual orientation?**

1. *Probe into why participant had preference for that survey question format.*
2. *If participant has no preference, probe into what the survey questions did correctly.*

**Can you describe your comfort level while answering each of these questions?**

**Imagine that you were being administered these questions as part of a health survey. Tell me about your relative comfort of answering these questions in the following ways:**

1: When an individual is asking you directly?

2: When an individual is asking you via video or web conferencing?

3: When responding to an electronic survey on a computer, phone, or tablet?

**What barriers, if any, did you experience while answering these questions?**

For each of the questions how would you feel about us including the prompt to either “choose one best answer” or “select all that apply?”?

Sexual Orientation Answer Choices:

**Now we’ll move on to the answer choices. Like we did with the question stems, please share your reactions when you read the answer choices. What did answer choices call to mind? You can also tell me what you like, dislike, or any changes you would make. We’ll go through each answer choice independently.**

- Straight, heterosexual
- Straight
- Heterosexual
- Lesbian, gay, same-gender attraction
- Lesbian
- Gay
- Same-gender attraction
- Bisexual
- Queer
- Pansexual
- Asexual, demisexual, graysexual
- Questioning/unsure
- Other: ___________
- Decline to state

**General comments about sexual orientation answer choices:**

**Was there any confusion when reviewing these answer choices?**

1. *If there was any confusion, ask what was the source of the confusion. Were there different interpretations of the answer choices that would change their answer?*
2. Did all of the words make sense to you? Did you understand the meaning of all of these words?

*If no, determine which words are unclear. Ask participant what they think the word or phrase means*.

**Some of the answer choices used commas to separate words while others used slashes. Tell me about any preference you had or would have for either grammatical presentation.**

1. *Probe into why participant had preference for that particular presentation*

**An answer choice that is being considered as an alternative to “lesbian, gay, same-gender attraction” is “lesbian, gay, homosexual”. Please describe your reactions to each of answer choices separately.**

1. *Probe into which answer choice the participant preferred*
2. *Probe into why participant had preference for that answer choice*

**An answer choice that is also being considered is “another: ______” as an alternative to “other: _____”. Please describe your reactions to both of these answer choices separately.**

1. *Probe into which answer choice the participant preferred*
2. *Probe into why participant had preference for that answer choice*

**SECTION 2: Ease of understanding gender identity questions and answer choices**

*Goals: To ascertain the participant’s understanding of question phrasing, question and answer choice vocabulary, and instructions.*

**Now we’ll repeat the same process for the gender identity question stems and answer choices. We’ll start with the question stems, please share your reactions when you read the question. What did this question stem call to mind? You can also tell me what you like, dislike, or any changes you would make. We’ll go through each question stem independently**

Gender Identity Question Stems:

1. What is your gender?
2. What is your gender identity?
3. What is your current gender?
4. What is your current gender identity?

**Was there any confusion when you were answering any of these questions?**

1. *If there was any confusion, ask what was the source of the confusion. Were there different interpretations of the question that would change their answer?*
2. Did all of the words make sense to you? Did you understand the meaning of all of these words?

*If no, determine which words are unclear. Ask participant what they think the word or phrase means*.

**Between the four questions, which did you prefer when asked about sexual orientation?**

1. *Probe into why participant had preference for that survey question*
2. *If participant has no preference, probe into what the survey questions did correctly*

**Can you describe your comfort level while answering each of these questions?**

**Imagine that you were being administered these questions as part of a health survey. Tell me about your relative comfort of answering these questions in the following ways:**

1: When an individual is asking you directly in person?

2: When an individual is asking you via video or web conferencing?

3: When responding to an electronic survey on a computer, phone, or tablet?

**What barriers did you experience while answering these questions?**

**For each of the questions how would you feel about us including the prompt to either “choose one best answer” or “select all that apply?”?**

Gender Identity Answer Choices:

**Now we’ll move on to the gender identity answer choices. Please share your reactions when you read the answer choices. What did they call to mind? You can also tell me what you like, dislike, or any changes you would make.**

- Female
- Male
- Woman
- Man
- Transgender female (MTF)
- Transgender male (FTM)
- Transgender woman (MTF)
- Transgender man (FTM)
- Gender non-conforming
- Questioning/unsure
- Other: ___________

**General comments about gender identity answer choices:**

**Was there any confusion when reviewing these answer choices?**

1. *If there was any confusion, ask what was the source of the confusion. Were there different interpretations of the answer choices that would change their answer?*
2. Did all of the words make sense to you? Did you understand the meaning of all of these words?

*If no, determine which words are unclear. Ask participant what they think the word or phrase means*.

**An answer choice that is being considered as an alternative to “gender non-conforming” is “gender non-binary”. Please describe your reactions to each of these wording options separately.**

1. *Probe into which answer choice the participant preferred.*
2. *Probe into why participant had preference for that answer choice.*

**An answer choice that is also being considered is “another: ______” as an alternative to “other: _____”. Please describe your reactions to both of these answer choices separately.**

1. *Probe into which answer choice the participant preferred*
2. *Probe into why participant had preference for that answer choice*

**SECTION 3: Ease of understanding the sex assigned at birth question and answer choices**

*Goals: To ascertain the participant’s understanding of question phrasing, question and answer choice vocabulary, and instructions.*

**Now we’ll repeat the process again for questions about “sex assigned at birth.” First, we will examine question stems and then answer choices. We’ll start with the question stems - please share your reactions when you read the question. What did this question stem call to mind? You can also tell me what you like, dislike, or any changes you would make.**

Sex Assigned at Birth Question Stems:

1. What sex were you assigned at birth?
2. What sex were you assigned at birth, meaning on your original birth certificate?

**Was there any confusion when you were answering this question?**

1. *If there was any confusion, ask what was the source of the confusion. Were there different interpretations of the question that would change their answer?*
2. Did all of the words make sense to you? Did you understand the meaning of all of these words?

*If no, determine which words are unclear. Ask participant what they think the word or phrase means*.

Sex Assigned at Birth Answer Choices:

**We’ll now move on to how sex assigned at birth answer choices. please share your reactions when you read the question. What did these answer choices call to mind? You can also tell me what you like, dislike, or any changes you would make.**

- **Female**
- **Male**
- **Intersex**

**General comments about sex assigned at birth answer choices:**

**Was there any confusion when reviewing these answer choices?**

1. *If there was any confusion, ask what was the source of the confusion. Were there different interpretations of the answer choices that would change their answer?*
2. Did all of the words make sense to you? Did you understand the meaning of all of these words?

*If no, determine which words are unclear. Ask participant what they think the word or phrase means*.

**That concludes our interview today. Do you have any questions before we finish?**

*[Pause to allow for participant to think of any questions]*

**Thank you so much for taking the time to participate! We will be sending you a gift card in the mail as a token of our appreciation. We asked you at the end of the survey to provide us with your mailing address but just to ensure that your gift card does not get lost would you mind giving it to me again?**

*[Record mailing address of the participant in the tracking spreadsheet]*

**Thank you for participating and enjoy the rest of your day!**

*[Exit out of Blue Jeans meeting window]*
